# Acetogenic Fermentation From Oxygen Containing Waste Gas

**DOI:** 10.3389/fbioe.2019.00433

**Published:** 2019-12-20

**Authors:** Teresa Mohr, Alba Infantes, Lars Biebinger, Pieter de Maayer, Anke Neumann

**Affiliations:** ^1^Technical Biology, Institute of Process Engineering in Life Science, Karlsruhe Institute of Technology, Karlsruhe, Germany; ^2^Faculty of Science, School of Molecular & Cell Biology, University of the Witwatersrand, Johannesburg, South Africa

**Keywords:** *Parageobacillus thermoglucosidasius*, *Clostridium ljungdahlii*, water-gas shift reaction, anaerobic acetate production, Wood-Ljungdahl pathway, syngas

## Abstract

The microbial production of bulk chemicals from waste gas is becoming a pertinent alternative to industrial strategies that rely on fossil fuels as substrate. Acetogens can use waste gas substrates or syngas (CO, CO_2_, H_2_) to produce chemicals, such as acetate or ethanol, but as the feed gas often contains oxygen, which inhibits acetogen growth and product formation, a cost-prohibitive chemical oxygen removal step is necessary. Here, we have developed a two-phase microbial system to facilitate acetate production using a gas mixture containing CO and O_2_. In the first phase the facultative anaerobic carboxydotroph *Parageobacillus thermoglucosidasius* was used to consume residual O_2_ and produce H_2_ and CO_2_, which was subsequently utilized by the acetogen *Clostridium ljungdahlii* for the production of acetate. From a starting amount of 3.3 mmol of CO, 0.52 mmol acetate was produced in the second phase by *C. ljungdahlii*. In this set-up, the yield achieved was 0.16 mol acetate/mol CO, a 63% of the theoretical maximum. This system has the potential to be developed for the production of a broad range of bulk chemicals from oxygen-containing waste gas by using *P. thermoglucosidasius* as an oxygen scrubbing tool.

## Introduction

The production of value-added chemicals, such as organic acids (e.g., acetate and succinate), glycerol derivatives (e.g., 2,3-butanediol and 1,3-propanediol) and alcohols (e.g., butanol, methanol, and ethanol) is still largely reliant on the use of fossil fuels as substrate (Hatti-Kaul et al., 2007; Zhang et al., 2017). Dwindling reserves and negative environmental effects associated with fossil fuel emissions underpin the necessity to develop novel inexpensive and environmentally friendly means of producing such chemicals. One potential alternative involves the use of synthesis gas (syngas) which consists primarily of hydrogen (H_2_), carbon dioxide (CO_2_), and carbon monoxide (CO) (Teixeira et al., 2018). Syngas can be produced from natural gas or coal as well as inexpensive feedstocks, such as lignocellulose (Barnard et al., 2010). Some microorganisms are capable of metabolizing the components from syngas into a wide range of chemical compounds, such as acetate, butanol, lactate, and ethanol (Liou et al., 2005; Drake et al., 2006; Köpke and Dürre, 2011; Daniell et al., 2012). Acetogens are microorganisms that are capable of producing acetyl-CoA out of two molecules of CO_2_ or CO via the Wood-Ljungdahl (W-L) pathway (Diekert and Wohlfarth, 1994). For example, *Clostridium ljungdahlii* can ferment CO_2_/H_2_ or CO/H_2_ via the W-L or Acetyl-CoA-pathway into acetyl-CoA. Further conversions lead to acetate as a main product and ethanol, butyrate, butanol and 2,3-butanediol in smaller amounts (Tanner et al., 1993; Köpke and Dürre, 2011). However, *C. ljungdahlii* and most other syngas fermenters are strict anaerobes, which limits the use of industrial waste gasses containing O_2_ (Liew et al., 2016). This can be linked to the oxygen sensitivity of many enzymes central to syngas fermentation pathways. For example, the key W-L pathway enzymes pyruvate-ferredoxin oxidoreductase (PFOR) and pyruvate formate lyase (PFL) are sensitive to very low levels of oxygen (Meinecke et al., 1989; Ragsdale and Wood, 1991; Bock et al., 1996; Brown et al., 1998; Becker et al., 1999; Imlay, 2009; Yang et al., 2009; Nakayama et al., 2013; Shen et al., 2017). In order to use microorganism to convert the components of industrial waste gas to a value-added product, O_2_ has to be removed first, a step which is cost prohibitive (Liew et al., 2016).

**Figure 1:**
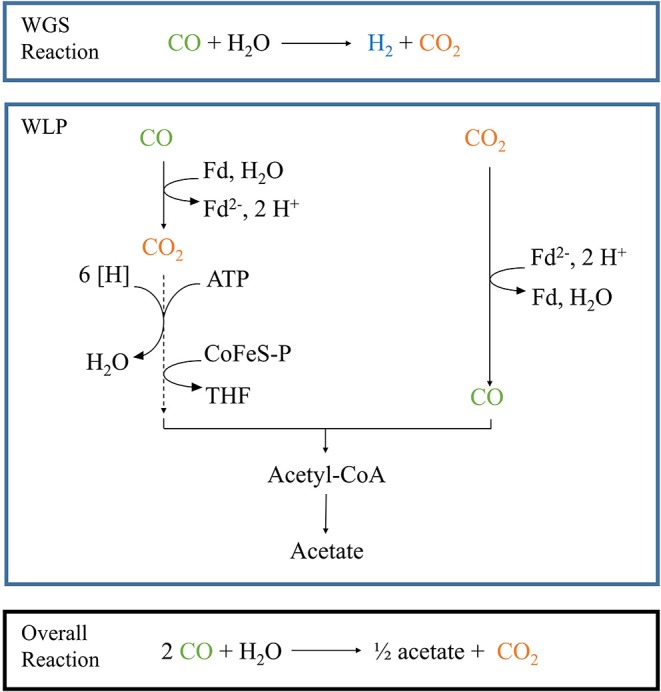
Schematic pathway of the combined WGS reaction and Wood-Ljungdahl pathway. The blue boxes depict each pathway separately, while the black box shows the result of the combined reactions in our particular set-up. Fd, oxidized ferredoxin; Fd^2−^, reduced ferredoxin; [H], reduction equivalents. Dotted lines depict a multiple-step reaction.

*Parageobacillus thermoglucosidasius* is a facultative anaerobic thermophile which is able to produce H_2_ and CO_2_ via the water-gas shift (WGS) when grown in the presence of a gas mixture consisting of CO and air (Mohr et al., 2018a,b) (Figure 1). Initially *P. thermoglucosidasius* supports its growth via aerobic respiration and once O_2_ is exhausted, it shifts to the anaerobic WGS pathway (Mohr et al., 2018a,b). This metabolic shift makes *P. thermoglucosidasius* a potential biological tool for the removal of O_2_ from syngas mixtures to be utilized in subsequent anaerobic production of value-added products. Furthermore, the CO_2_ and H_2_ produced by this organism can serve as substrates for the production of chemical compounds, such as ethanol, butanol, butyric acid, acetate and methane (Drake et al., 2006; Köpke et al., 2010; Liew et al., 2016).

In the present study we have undertaken sequential fermentation with *P. thermoglucosidasius* and *C. ljungdahlii* and demonstrate the potential of using an O_2_-depleting facultative anaerobe to facilitate the anaerobic production of value-added products from an artificial waste gas, a gas mixture containing both CO and O_2_.

## Materials and Methods

### Microorganisms and Media

*P. thermoglucosidasius* DSM 6285 and *Clostridium ljungdahlii* DSM 13528^T^ were obtained from the Deutsche Sammlung von Mikroorganismen und Zellkulturen (DSMZ, Braunschweig, Germany).

*P. thermoglucosidasius* DSM 6285 was cultivated in mLB (modified Luria-Bertani) medium (g/l): tryptone (10), yeast extract (5), NaCl (5); 1.25 ml/l NaOH (10% w/v), and 1 ml/l of each of the filter-sterilized stock solutions 1.05 M nitrilotriacetic acid, 0.59 M MgSO_4_·7H_2_O, 0.91 M CaCl_2_·2H_2_O, and 0.04 M FeSO_4_·7H_2_O (Zeigler, 2001). A first (20 ml mLB medium) pre-culture was grown for 24 h and a second (20 ml mLB medium) pre-culture was inoculated to an absorbance (OD_600_) of 0.1 from the first pre-culture and incubated for 4 h. Both pre-cultures were grown aerobically at 60°C and 120 rpm (Infors Thermotron, Infors AG, Bottmingen, Switzerland) in 20 ml mLB. Serum bottles (Glasgerätebau Ochs, Bovenden, Germany), closed with gas-tight butyl rubber stoppers and secured with an aluminum seal were used (Carl Roth + Co. KG, Karlsruhe, Germany).

*Clostridium ljungdahlii* DSM 13528^T^ was pre-cultured in modified GA-based medium (Groher and Weuster-Botz, 2016) containing (g/l): 2-(N-morpholino) ethansulfonic acid (MES) (20), / NH_4_Cl (1), KCl (0.3), KH_2_PO_4_ (0.23), MgSO_4_·7H_2_O (0.5), NaCl (2.25), yeast extract (2), CaCl_2_·2H_2_O (0.15), and resazurin (0.001). The pH of the medium was adjusted to 6.0 with KOH, and distributed in serum bottles. These were then closed with gas-tight butyl rubber stoppers and secured with an aluminum seal, and anaerobized. The anaerobization process was performed as follows: a needle, which was connected to a vacuum/gas line, was inserted through the septum; then, vacuum was applied to a final pressure of 10 psi (absolute), holding for 40 s, followed by pressurizing the bottles to 30 psi (absolute) using a gas mixture containing 20 vol-% carbon dioxide in nitrogen (Air Liquide, France). Following this, vacuum was applied again, and the whole process was repeated for 20 cycles. The bottles were then autoclaved closed. After autoclaving, the following solutions were added to the bottles (g/L): cysteine HCl·H_2_O (1), fructose (10); 1 ml/l of trace element solution (mg/L): FeSO_4_·7H_2_O (4000), Na_2_SeO_3_·5H_2_O (3), Na_2_WO_4_·2H_2_O (4), FeCl_2_·4H_2_O (3000), ZnCl_2_ (140), MnCl_2_·4H_2_O (200), H_3_BO_3_ (12), CoCl_2_·6H_2_O (380), CuCl_2_·2H_2_O (4), NiCl_2_·6H_2_O (48), Na_2_MoO_4_·2H_2_O (72), and 10 ml/l of vitamin solution (mg/L): biotin (4), folic acid (4), pyridoxine (20), thiamine-HCl·2H_2_O (10), riboflavin (10), nicotinic acid (10), calcium pantothenate (10), cobalamin (0.2), 4-aminobenzoic acid (10), and lipoic acid (10). To ensure sterility and anaerobic conditions, all additions to the autoclaved bottles were done using sterile syringes and needles, and piercing through the septum. All stock solutions were prepared and anaerobized as described above.

For pre-cultivation of *C. ljungdahlii* a glycerol stock (1 ml) was transferred anaerobically to a serum bottle containing 50 ml of anaerobic, sterile GA medium (prepared as above) and incubated for 48 h. A total of 5 ml of the latter culture was transferred anaerobically to 50 ml of fresh GA medium and cultivated at 37°C and 120 rpm for 24 h. The latter step was repeated to generate the inoculum for the sequential culture. The glycerol stocks were prepared in sterile, anaerobic hungate-type tubes which were closed with gas-tight rubber septa and secured with a perforated screw cap. In order to anaerobically transfer the glycerol stock and to inoculate the following cultures, sterile syringes and needles were used. The withdrawal of the liquid and its addition to the following serum bottle was performed by piercing through the septum.

### Experimental Set Up

Stoppered serum flasks (250 ml), gas-tight and prepared as above, containing 50 ml of modified Luria Bertani (mLB) medium and with an initial gas atmosphere of CO and air (50:50 ratio) were inoculated with 1 ml of second pre-culture of *P. thermoglucosidasius* and cultivated for 70 h at 60°C and 120 rpm. Subsequently, 5 ml of the *C. ljungdahlii* pre-culture (OD_600_ = 2.5) was added to the *P. thermoglucosidasius* culture. Immediately before inoculating with *C. ljungdahlii*, 50 μl of GA trace elements, to the same final concentration as the GA medium, were added to each bottle to ensure that all elements necessary for the growth of *C. ljungdahlii* were present. Incubation of the *P. thermoglucosidasius*/*C. ljungdahlii* cultures were performed at 37°C and 120 rpm. The experiments were performed in quadruplicate for a duration of 240 h.

### Analytical Methods

Growth was routinely monitored by taking 1 ml culture samples twice per day and performing absorbance (OD_600_) measurements using an Ultrospec 1100 pro spectrophotometer (Amersham Biosciences, USA). Acetate concentrations were similarly monitored using the Roche Yellow line enzymatic assay (Hoffmann- La Roche, Switzerland). To measure the gas composition in the bottles at each sampling point, a 5 ml gas sample was withdrawn with a syringe from the headspace of the bottle. The bottles were kept at the incubating temperature for the specific microorganism by means of a water bath. The sample was then immediately injected into a 300 Micro GC gas analyzer (Inficon, Bad Ragaz, Switzerland) with columns Molsieve and PLOT Q. Throughout the total analysis time of 180 s, the temperature was maintained constantly at 80°C.

Pressure was measured before and after sample taking using a manometer (GDH 14 AN, Greisinger electronic, Regenstauf, Germany). Gas composition was calculated using the ideal gas law as previously described (Mohr et al., 2018a). The acetate yield was calculated based on Bengelsdorf et al. (2013).

## Results

### Pre-culturing With *P. thermoglucosidasius* Supports the Anaerobic Growth of *C. ljungdahlii*

**Figure 2:**
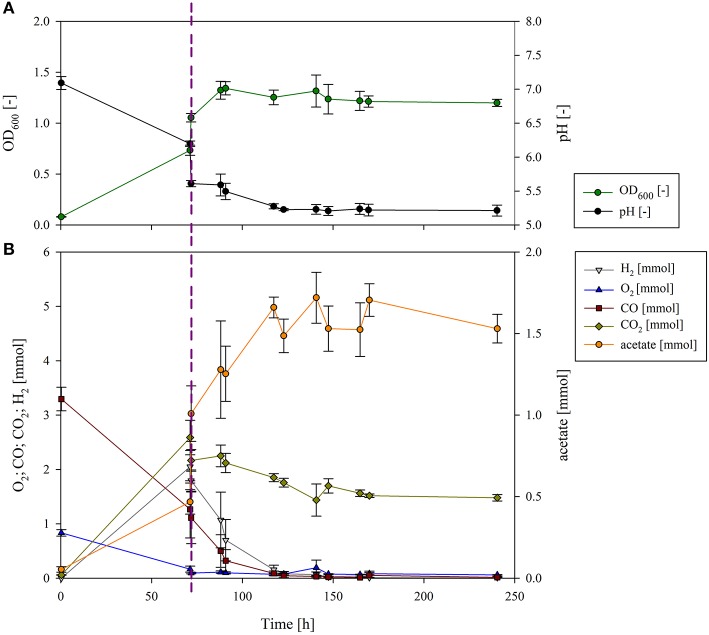
Growth and pH **(A)** and gas composition and acetate production **(B)** of the sequential cultivation of *P. thermoglucosidasius* and *C. ljungdahlii*. The dotted line presents the inoculation of *C. ljungdahlii*. **(A)** The measured OD_600_ (dark green) increased after 70 h, and at the same time the pH (black) decreased due to the inoculation with *C. ljungdahlii*. Growth continued until 93 h (23 h after inoculation with the second organism), and then it plateaued. As a result of the metabolic activity, the culture broth was acidified to a pH of 5.2. **(B)** O_2_ (blue) had already been consumed before the second phase, but some CO (dark red) was still left. After inoculation with *C. ljungdahlii*, CO_2_ (olive), and H_2_ (gray) did not accumulate any further, since they were used as building blocks by *C. ljungdahlii* to produce acetate (orange).

In the first phase of the sequential fermentation *P. thermoglucosidasius* was grown in 50 ml modified Luria Bertani (mLB) medium with an initial gas atmosphere of CO and air (50:50) (Figure 2). After 70 h, when all O_2_ was consumed, the culture reached an absorbance (OD_600_) of 0.732 ± 0.027 and pH of 6.21 ± 0.04 (Figure 2A). Previously we have observed that when the O_2_ is consumed, the growth of *P. thermoglucosidasius* also plateaus (Mohr et al., 2018a,b). To ensure that the increase of OD_600_ and acetate during the second phase is not due to *P. thermoglucosidasius* on its own, a control experiment without the addition of *C. ljungdahlii* was conducted (Figure S1). When *C. ljungdahlii* was added to the *P. thermoglucosidasius* culture 70 h after the first phase, the *P. thermoglucosidasius*/*C. ljungdahlii* sequential culture reached a maximum absorbance of 1.316 ± 0.157 ~23 h after the latter culture was added (Figure 2A). This indicates that the strict anaerobe *C. ljungdahlii* is able to grow in the medium after *P. thermoglucosidasius* exhausts the O_2_ from the gas atmosphere. The medium pH dropped drastically once *C. ljungdahlii* was added, from a pH of 6.20 ± 0.04 pre-addition to a pH of 5.61 ± 0.05 post-addition of the latter strain (Figure 2A). However, the pH continued to decline throughout the experiment, which can be correlated to active metabolism and acetate production by *C*. *ljungdahlii*.

### Sequential Cultivation With *P. thermoglucosidasius* and *C. ljungdahlii* Facilitates Acetate Production

In the post-aerobic phase *P. thermoglucosidasius* consumed 2.050 ± 0.117 mmol of CO, while 2.055 ± 0.023 and 2.646 ± 0.147 mmol of H_2_ and CO_2_ were produced via the WGS, respectively. Here, an equimolar conversion of CO to H_2_ was achieved. Subsequently, both H_2_ and CO decreased rapidly, being exhausted ~83 h after *C. ljungdahlii* was added. Similarly, CO_2_ decreased, although 1.479 ± 0.058 mmol CO_2_ were left at the end of the cultivation (after 240 h), due to the fact that 2 moles of H_2_ are needed per mol of CO_2_ as per the stoichiometry of the W-L pathway: 2 CO_2_ + 4 H_2_ → CH_3_COOH + 2 H_2_O (Ragsdale, 2008).

The decrease in the amount of these three gasses correlated with an increase in the amount of acetate. Some acetate (0.47 ± 0.07 mmol) was already observed during the first phase. This may be linked to mixed acid fermentation by *P. thermoglucosidasius* (Hussein et al., 2015). However, when *P. thermoglucosidasius* was cultivated on its own, no further increase in acetate was observed (Figure S1). The addition of *C. ljungdahlii* resulted in a spike in acetate (1.01 ± 0.17 mmol—an increase of 0.54 ± 0.22 mmol). This is associated with acetate production by *C. ljungdahlii* in the pre-culture in GA medium containing fructose as carbon source (Tirado-Acevedo et al., 2011). To shorten the time of inoculation of *C. ljungdahlii*, a washing step was not performed to avoid any potential lag phase due to stressing of the cells. Nevertheless, the amount of acetate increased concomitantly with H_2_, CO and CO_2_ consumption during the second phase, reaching a final amount of 1.53 ± 0.09 mmol of acetate. The acetate produced exclusively by *C. ljungdahlii* was, therefore, 0.52 mmol. This suggests that in the absence of additional exogenous carbon sources *C. ljungdahlii* could successfully use the H_2_ and CO_2_ produced by *P. thermoglucosidasius* as building blocks for acetate via the W-L pathway.

From the WLP, the theoretical maximum yield is 0.25 mol acetate/mol CO (Bengelsdorf et al., 2013). Considering the initial amount of CO in the bottles, 3.3 ± 0.216 mmol in average, a total theoretical maximum of 0.8 mmol of acetate could have been produced. The yield of acetate in the *C. ljungdahlii* phase in this study was 0.16 mol acetate/mol CO, achieving a 63% of the theoretical maximum.

## Discussion and Conclusion

Microbial conversion of syngas into value-added chemicals may provide a sustainable and cost-effective alternative to current industrial strategies. However, most known syngas fermenters are strict anaerobes, which impacts the use of syngas sources which contain even low concentrations of O_2_. Besides, very few acetogens have been shown to tolerate only trace amounts of O_2_ (Karnholz et al., 2002; Takors et al., 2018). As such, expensive and often environmentally unfriendly O_2_ removal steps are necessary to facilitate effective syngas bioconversion (Heijstra et al., 2017). Here we have demonstrated that the facultative anaerobe *P. thermoglucosidasius* provides a biological means for the removal of toxic concentrations of O_2_, which allowed for the subsequent growth of the strict anaerobe *C. ljungdahlii*. Moreover, the production of H_2_ and CO_2_ by *P. thermoglucosidasius* via the WGS reaction provides the building blocks for the synthesis of acetate by *C. ljungdahlii* via the W-L pathway.

The utilization of a thermophile in the first phase of this process presents some additional advantages in that hot flue gasses resulting from industrial processes will not need to be cooled down to such a great extent. Most pertinently, the consumption of CO enables a near stoichiometric conversion of CO to H_2_ and CO_2_, without CO being lost in biomass formation (Mohr et al., 2018a) unlike in other CO-oxidizing organism, where CO is also used for biomass formation (O_2_ + 2.19 CO → 1.83 CO_2_ + 0.36 cell carbon) (Ragsdale, 2004). In this study the consumed CO during the first phase was completely converted to H_2_ and CO_2_ by *P. thermoglucosidasius* without CO being used for acetate production or growth. Hence, more substrate for the acetogenesis is available. With the sequential cultivation, a total amount of 1 mmol of acetate was produced. From this, the amount of acetate derived from the initial CO amounted to 0.52 mmol, which represents a 63% of the maximum theoretical yield. The overall yield of the established sequential culture is thus higher than by using other CO metabolizing organisms (King and Weber, 2007).

The sequential fermentation system presented here may thus serve as the basis for establishing as a cost-effective and environmentally friendly methodology for the production of value-added chemicals where it circumvents some of the pitfalls of working with strict anaerobic syngas fermenters while simultaneously linking the fermentative pathways of different taxa for the production of value-added chemicals by a second organism (Figure 1) (Takors et al., 2018). Future research will optimize the set-up and evaluate the application of this sequential fermentation with *P. thermoglucosidasius* and other mesophilic and thermophilic anaerobic bacteria for the production of a wide variety of bulk chemicals.

## Data Availability Statement

The datasets generated for this study are available on request to the corresponding author.

## Author Contributions

TM and AI planned the experiments, collected and analyzed the experimental data. LB conducted his Bachelor thesis under the supervision of TM and AI. AN and PM substantially contributed to the conception and design of the experiments. TM, AI, and PM drafted the manuscript. All authors read and approved the final version of the manuscript.

### Conflict of Interest

The authors declare that the research was conducted in the absence of any commercial or financial relationships that could be construed as a potential conflict of interest.
