# Supplementary figures and images for: Acetogenic Fermentation From Oxygen Containing Waste Gas
Source: Front Bioeng Biotechnol. 2019 Dec 20;7:433. doi: 10.3389/fbioe.2019.00433 (PMC6932952; doi:10.3389/fbioe.2019.00433)

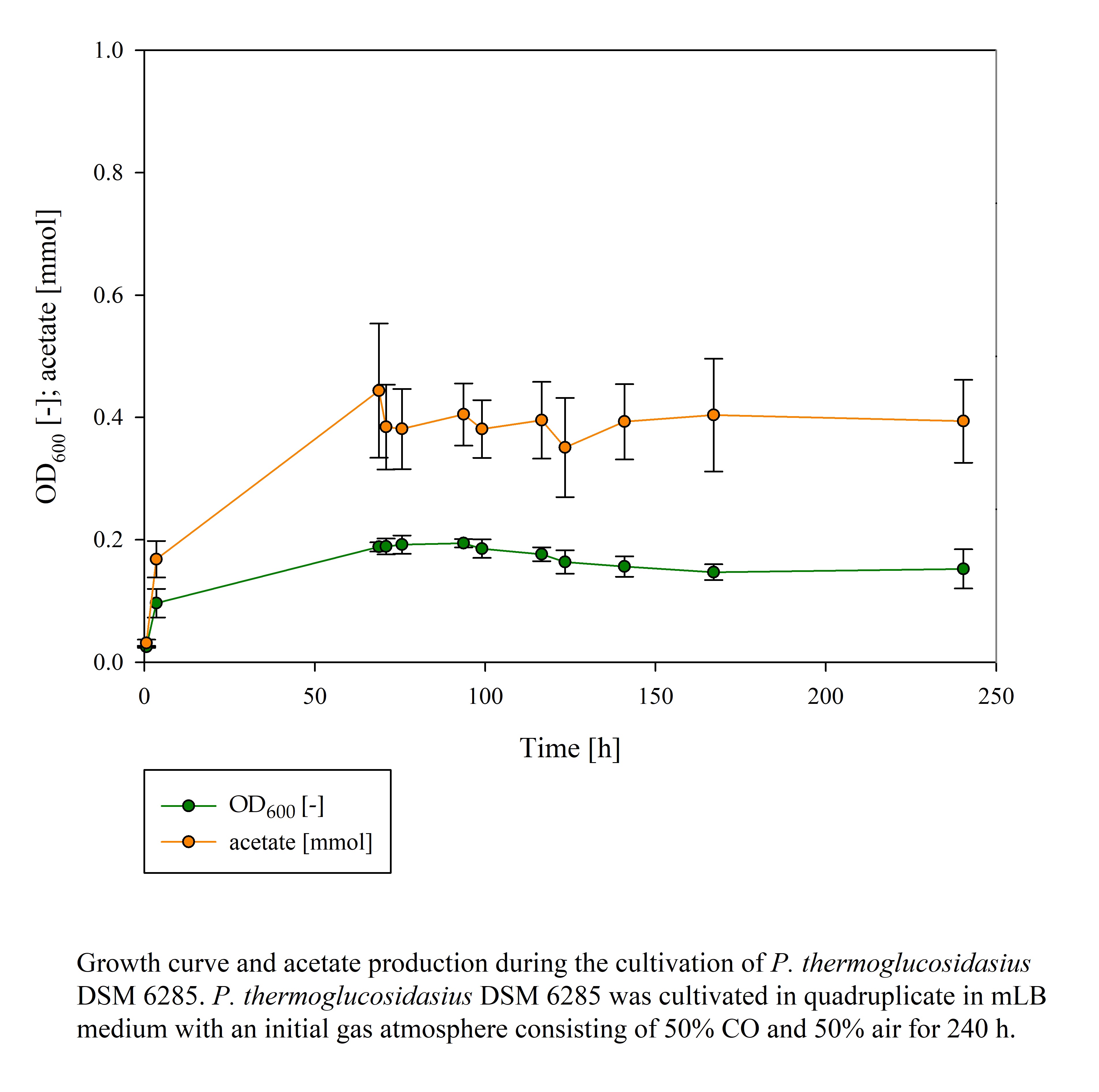

Supplement: Supplementary file 1 [file Image_1.JPEG]
